# Supplementary material for: The causal relationship between the human gut microbiota and pyogenic arthritis: a Mendelian randomization study
Source: Front Cell Infect Microbiol. 2024 Nov 26;14:1452480. doi: 10.3389/fcimb.2024.1452480 (PMC11629706; doi:10.3389/fcimb.2024.1452480)
Supplement: Supplementary file 1 [file DataSheet1.zip › mendelian test/ebi-a-GCST90027529.csv_leave_one_out.pdf]

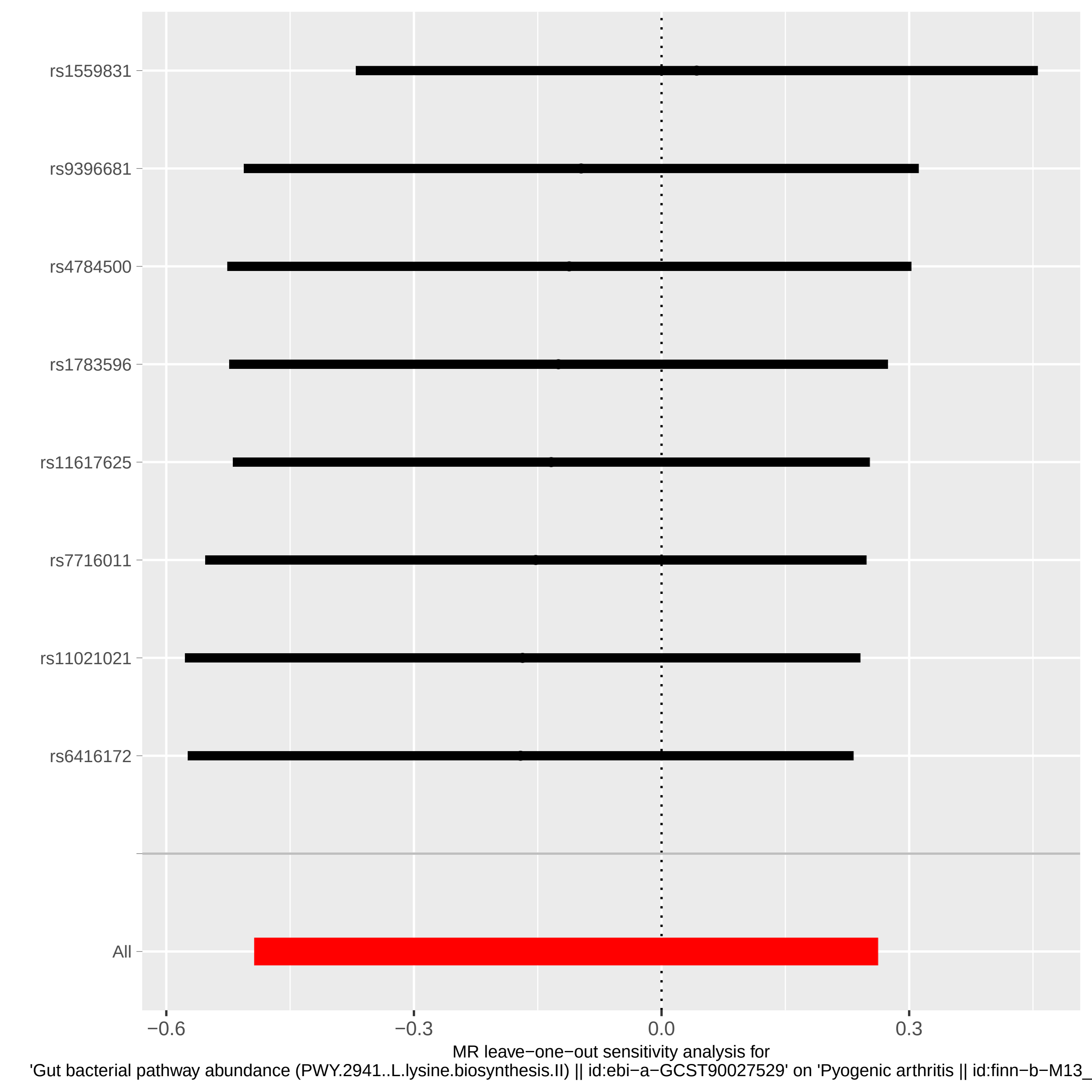

rs1559831
rs9396681
rs4784500
rs1783596
rs11617625
rs7716011
rs11021021
rs6416172
All
−0.6
−0.3
0.0
0.3
MR leave−one−out sensitivity analysis for
'Gut bacterial pathway abundance (PWY.2941..L.lysine.biosynthesis.II) || id:ebi−a−GCST90027529' on 'Pyogenic arthritis || id:finn−b−M13_
